# Supplementary material for: Liver transplant recipients with polycystic liver disease have longer waiting times but better long-term clinical outcomes than those with liver disease due to other causes: A retrospective cross-sectional study
Source: PLoS One. 2024 Jan 2;19(1):e0294717. doi: 10.1371/journal.pone.0294717 (PMC10760649; doi:10.1371/journal.pone.0294717)
Supplement: S1 Checklist — (DOCX) [file pone.0294717.s001.docx]

STROBE Statement—checklist of items that should be included in reports of observational studies

|  | Item No. | Recommendation | Page  No. | Relevant text from manuscript |
| --- | --- | --- | --- | --- |
| **Title and abstract** | 1 | (*a*) Indicate the study’s design with a commonly used term in the title or the abstract | 1 | Liver transplant recipients with polycystic liver disease have longer waiting times but better clinical outcomes than those with liver disease due to other causes: A retrospective cross-sectional study. |
|  |  | (*b*) Provide in the abstract an informative and balanced summary of what was done and what was found | 3-4 | A retrospective cross-sectional analysis of patients receiving liver transplantation between 2010 and 2017 was performed using the NHS blood and transplantation database. This database contains the demographic, clinical parameters, indication for transplantation and follow-up of all patients in UK-based transplant centres. Basic statistics was performed using SPSS version 27. |
| Introduction | | | |  |
| Background/rationale | 2 | Explain the scientific background and rationale for the investigation being reported | 5-6 | *Please see information included in the introduction.* |
| Objectives | 3 | State specific objectives, including any prespecified hypotheses | 6 | The primary objective of this study is to compare the waiting times and outcomes of patients with PLD and those with other indications for liver transplantation prior to the change in the allocation model for the UK. The secondary objective is to identify any variation in liver transplantation rates for people with PLD between UK based transplant centres. |
| Methods | | | |  |
| Study design | 4 | Present key elements of study design early in the paper | 7 | A retrospective cross-sectional study was performed from the NHSBT Liver Transplant dataset of all patients undergoing primary liver transplantation in the UK between 01/01/2010 to 31/12/2017. |
| Setting | 5 | Describe the setting, locations, and relevant dates, including periods of recruitment, exposure, follow-up, and data collection | 7 | A retrospective cross-sectional study was performed from the NHSBT Liver Transplant dataset of all patients undergoing primary liver transplantation in the UK between 01/01/2010 to 31/12/2017. |
| Participants | 6 | (*a*) *Cohort study*—Give the eligibility criteria, and the sources and methods of selection of participants. Describe methods of follow-up  *Case-control study*—Give the eligibility criteria, and the sources and methods of case ascertainment and control selection. Give the rationale for the choice of cases and controls  *Cross-sectional study*—Give the eligibility criteria, and the sources and methods of selection of participants | 7 | Eligibility criteria:   - Age ≥ 18 years - Recipient of a liver allograft, liver alone or combined liver-kidney, between 01/01/2010 and 31/12/2017 - Transplantation at a UK based transplant centre - Not transplanted for a super-urgent indication |
|  |  | (*b*) *Cohort study*—For matched studies, give matching criteria and number of exposed and unexposed  *Case-control study*—For matched studies, give matching criteria and the number of controls per case | NA | NA – not matched study |
| Variables | 7 | Clearly define all outcomes, exposures, predictors, potential confounders, and effect modifiers. Give diagnostic criteria, if applicable | 7 | Variables included the year of transplantation, CLD/HCC or VS category, cause of liver disease, waiting time, transplant centre, recipient details (gender, age and ethnicity), creatinine at the time of listing, UKELD, Model for End-stage Liver Disease (MELD) score and survival. Survival rates were informed by patient death (patient survival), allograft failure (allograft survival) or either combined (transplant failure). |
| Data sources/ measurement | 8* | For each variable of interest, give sources of data and details of methods of assessment (measurement). Describe comparability of assessment methods if there is more than one group | 7 | The primary liver disease diagnosis is reported by the referring transplant centre. Diagnosis of PLD may be based on genetic analysis or the identification of multiple liver cysts on liver imaging (Reynolds criteria). |
| Bias | 9 | Describe any efforts to address potential sources of bias | 7 | Before and during data analysis, the researchers were blinded to the participant’s diagnosis and the transplanting centre in order to reduce bias. |
| Study size | 10 | Explain how the study size was arrived at | 7 | Total number of patients registered to NHSBT Liver Transplant dataset. |

Continued on next page

| Quantitative variables | 11 | Explain how quantitative variables were handled in the analyses. If applicable, describe which groupings were chosen and why |  | Quantitative variables were analysed in their continuous form with no categorisation performed. |
| --- | --- | --- | --- | --- |
| Statistical methods | 12 | (*a*) Describe all statistical methods, including those used to control for confounding | 8 | Statistical analysis was performed using SPSS 2020 and Kaplan-Meier survival analysis. |
|  |  | (*b*) Describe any methods used to examine subgroups and interactions | 8 | Sub-group analysis was performed using ANOVA to assess the difference between liver alone and liver-kidney allografts. |
|  |  | (*c*) Explain how missing data were addressed | 8 | Any data subjects with incomplete demographic, primary disease, list type or survival data were excluded. |
|  |  | (*d*) *Cohort study*—If applicable, explain how loss to follow-up was addressed  *Case-control study*—If applicable, explain how matching of cases and controls was addressed  *Cross-sectional study*—If applicable, describe analytical methods taking account of sampling strategy | 8 | Statistical analysis was performed using SPSS 2020 and Kaplan-Meier survival analysis. |
|  |  | (*e*) Describe any sensitivity analyses | 8 | We compared recipient characteristics using the Chi^2^ test for categorical variables and the independent t-test for continuous variables. |
| Results | | | | |
| Participants | 13* | (a) Report numbers of individuals at each stage of study—eg numbers potentially eligible, examined for eligibility, confirmed eligible, included in the study, completing follow-up, and analysed | 8 | *See Fig 1* |
|  |  | (b) Give reasons for non-participation at each stage | 8 | *See Fig 1* |
|  |  | (c) Consider use of a flow diagram | 8 | *See Fig 1* |
| Descriptive data | 14* | (a) Give characteristics of study participants (eg demographic, clinical, social) and information on exposures and potential confounders | 9 | *See Table 1* |
|  |  | (b) Indicate number of participants with missing data for each variable of interest | 8-9 | *See Fig 1 and Table 1* |
|  |  | (c) *Cohort study*—Summarise follow-up time (eg, average and total amount) |  | NA – not cohort study |
| Outcome data | 15* | *Cohort study*—Report numbers of outcome events or summary measures over time | NA | NA – not cohort study |
|  |  | *Case-control study—*Report numbers in each exposure category, or summary measures of exposure | NA | NA – not case-control study |
|  |  | *Cross-sectional study—*Report numbers of outcome events or summary measures | *8* | *See Fig 1* |
| Main results | 16 | (*a*) Give unadjusted estimates and, if applicable, confounder-adjusted estimates and their precision (eg, 95% confidence interval). Make clear which confounders were adjusted for and why they were included | 9-13 | *See Tables 1-5* |
|  |  | (*b*) Report category boundaries when continuous variables were categorized | NA | NA – no categorisation of variables |
|  |  | (*c*) If relevant, consider translating estimates of relative risk into absolute risk for a meaningful time period | NA | NA – no inclusion of relative risk |

Continued on next page

| Other analyses | 17 | Report other analyses done—eg analyses of subgroups and interactions, and sensitivity analyses | 13 | *See Table 5* |
| --- | --- | --- | --- | --- |
| Discussion | | | | |
| Key results | 18 | Summarise key results with reference to study objectives | 14 | Patients with PLD wait 3 times longer for liver transplantation than those with non-PLD CLD/HCC and other variant syndromes… Our study demonstrates that there are comparable survival rates, patient and allograft, for patients with PLD and non-PLD indications for transplantation at 30 days and 1 year. |
| Limitations | 19 | Discuss limitations of the study, taking into account sources of potential bias or imprecision. Discuss both direction and magnitude of any potential bias | 16 | We report transplantation rates prior to the updated listing policy in 2018 for people with and without PLD in UK-based transplant centres. One weakness of this study is that we are unable to comment on the characteristics of patients not listed for transplantation or those on the waiting list. Although there is overall a high level of complete data; there is incomplete reporting of intraoperative death (87.9% complete), patient death (90.7% complete) and graft failure (99.6% complete). |
| Interpretation | 20 | Give a cautious overall interpretation of results considering objectives, limitations, multiplicity of analyses, results from similar studies, and other relevant evidence | 16 | Patients with PLD have a longer waiting time to transplantation compared to those with other indications for liver transplantation but better long-term patient and liver allograft survival rates. This is independent of the type of list at registration, chronic liver disease/hepatocellular carcinoma or variant syndrome list. There is significant variation in the numbers of patients with PLD undergoing liver transplantation at different UK-based transplant centres. |
| Generalisability | 21 | Discuss the generalisability (external validity) of the study results | 15 | Although organ allocation policies vary between different countries limiting generalisability this study adds to the existing literature from other countries demonstrating similar outcomes. |
| Other information | |  | | |
| Funding | 22 | Give the source of funding and the role of the funders for the present study and, if applicable, for the original study on which the present article is based | 17 | MG received financial support from NIHR for an Academic Clinical Fellowship. |

*Give information separately for cases and controls in case-control studies and, if applicable, for exposed and unexposed groups in cohort and cross-sectional studies.

**Note:** An Explanation and Elaboration article discusses each checklist item and gives methodological background and published examples of transparent reporting. The STROBE checklist is best used in conjunction with this article (freely available on the Web sites of PLoS Medicine at http://www.plosmedicine.org/, Annals of Internal Medicine at http://www.annals.org/, and Epidemiology at http://www.epidem.com/). Information on the STROBE Initiative is available at www.strobe-statement.org.
