# Supplementary figures and images for: Liver transplant recipients with polycystic liver disease have longer waiting times but better long-term clinical outcomes than those with liver disease due to other causes: A retrospective cross-sectional study
Source: PLoS One. 2024 Jan 2;19(1):e0294717. doi: 10.1371/journal.pone.0294717 (PMC10760649; doi:10.1371/journal.pone.0294717)

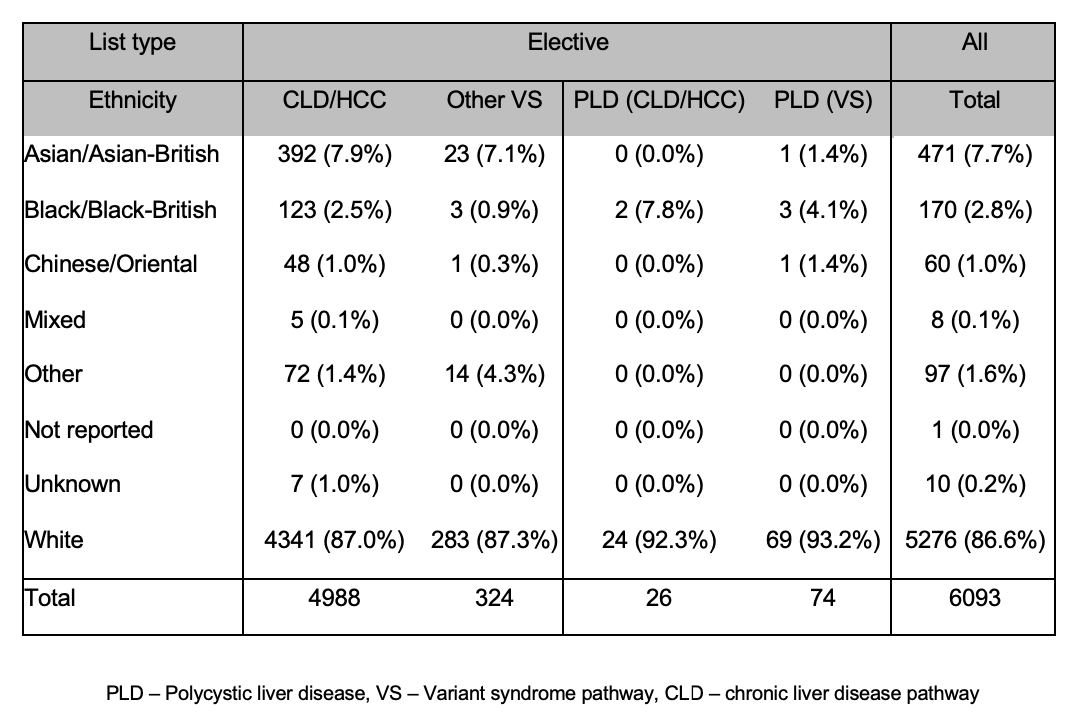

Supplement: S1 Table — (PNG) [file pone.0294717.s002.png]

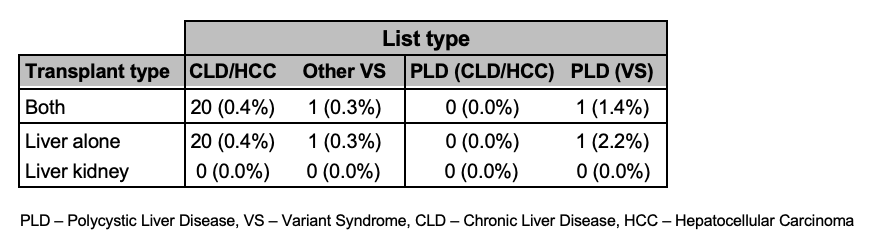

Supplement: S2 Table — (PNG) [file pone.0294717.s003.png]
