# Supplementary material for: Liver transplant recipients with polycystic liver disease have longer waiting times but better long-term clinical outcomes than those with liver disease due to other causes: A retrospective cross-sectional study
Source: PLoS One. 2024 Jan 2;19(1):e0294717. doi: 10.1371/journal.pone.0294717 (PMC10760649; doi:10.1371/journal.pone.0294717)
Supplement: Attachment — Submitted filename: PLD response to authors.docx [file pone.0294717.s004.docx]

Dear Editor-in-Chief,

Thank you for the opportunity to revise and resubmit our manuscript entitled “Liver transplant recipients with polycystic liver disease have longer waiting times but better long-term clinical outcomes than those with liver disease due to other causes: A retrospective cross-sectional study”, for publication in *PLOS One*.

We would like to thank the reviewers and editors for the time and effort spent reviewing our manuscript. The comments and suggestions have allowed us to improve both the content and accessibility of our manuscript for the wider transplant community.

We have submitted our detailed response to reviewer comments, and a revised version of the manuscript. In order to aid the reader in comparing the PLD and non-PLD groups we have increased the overall data granularity. The abstract has also been updated to reflect this information. Unfortunately, we do not currently have access to the data for patients on the transplant waiting list who have not yet received a liver allograft.

We are grateful for your consideration of the revised manuscript.

Yours sincerely,

Dr Matthew Gittus

**Journal Requirements:**

When submitting your revision, we need you to address these additional requirements.

1. Please ensure that your manuscript meets PLOS ONE's style requirements, including those for file naming.

*Thank you for this recommendation and the link to your guideline on style requirements.*

2. PLOS requires an ORCID iD for the corresponding author in Editorial Manager on papers submitted after December 6th, 2016. Please ensure that you have an ORCID iD and that it is validated in Editorial Manager.

*Thank you, the ORCID iD has been added to the submission on the Editorial Manager.*

3. Please ensure that you refer to Figure 6 in your text as, if accepted, production will need this reference to link the reader to the figure.

*Thank you for highlighting this error. It has been adjusted in the revised manuscript.*

**Additional Editor Comments:**

PONE-D-23-17780 The Editor reviewed the manuscript, and agreed with the Reviewer # 1. However, additional consideration need to be addressed in the manuscript.  The Abstract needs to be enriched with the actual specific data. The Results section in the Abstract is descriptive. Please provide specific numbers for the waiting time and for the survival. It is also confusing in precision of describing patients who underwent a combined liver-kidney transplantation. Out of 100 recipients with PLD how many had liver and how many had liver-kidney transplantation? Are there their waiting times different? What about their graft and patient survival times at 30 days, one and five years? The last section should be entitled conclusions.

*Thank you for your recommendation. We have increased the amount of data included in the abstract in the results section. The data points suggested for inclusion have been specifically included in the revised abstract. As requested the last section has been entitled “conclusions”.*

The authors need to explain better the reasons for the graft failure in PLD group vs. non-PLD group as this point is very interesting. The possible difference between two groups may be related to the recurrence of the disease in non-PLD group, but this needs to be better documented.  Please address the question about the mortality on the waiting list for PLD group as this is especially interesting with an extended waiting time for the transplant. As suggested by the Reviewer # 1, please present more extensively about the waiting time mortality in PLD and non-PLD groups: additional table may be helpful.

*Thank you for this recommendation, it has been addressed through improving the data granularity in all tables included in the manuscript. As you suggested the main difference between the non-PLD and PLD groups is related to recurrence of disease in the non-PLD group. This is important as the PLD group cannot have recurrence due to the genetic basis for their condition. The updated section of the results section is located on lines 234-239 of the revised manuscript. Unfortunately, we do not currently have the data in regard to patients on the waiting list so cannot provide any analysis on mortality whilst on the waiting list. We are planning to request this data for a future study.*

Please address the comments by the Reviewer # 1:

Lines 211-213: Discussed overall allograft failure in the data. The graph shows that allograft failure is much less in PCLD and that there is no recurrent disease in these patients, which is the leading reason for allograft failure in non-PCLD transplants. I recommend highlighting this point in your text.

*Thank you for your recommendation, we agree that this point needs to made more clearly in the manuscript. This has been highlighted on lines 333-342 which has been revised to include the comment that there is no recurrent disease in PLD patients due to the nature of their disease.*

Lines 250-253: It would be interesting to see data on wait list mortality for these patients, especially in light of PCLD-VS patients having the longest wait time. If you have that data, I would recommend presenting it in this paper.

*Thank you, we agree that this would be a very interesting approach to highlight the potential harm from increased waiting times in the PLD group. We are planning to request this data for a future study.*

They report several interesting findings. They find that patients transplanted for PCLD have a higher 5 year survival but a longer wait list time. They found that PCLD patients listed for variant syndrome had the longest waiting time- longer than other variant syndrome patients and longer than PCLD listed with HCC or CLD. They also find that there is a significant difference between transplant centers, suggesting that the VS criteria in particular can lead to variations in interpretation. This study provides insight into the transplant outcomes for patients with PCLD.  All changes must be explained and marked in the revised vesrion.

*Thank you for these comments. Based on your comments we have improved the data granularity for this section by revising table 5 and including table 6. This hopefully indicates the potential role that transplantation rates from the VS list can have on variation between centres. This has also been indicated in the discussion on lines 345-347.*
